# Collection by trained pediatricians or parents of mid-turbinate nasal flocked swabs for the detection of influenza viruses in childhood

**DOI:** 10.1186/1743-422X-7-85

**Published:** 2010-04-30

**Authors:** Susanna Esposito, Claudio G Molteni, Cristina Daleno, Antonia Valzano, Claudia Tagliabue, Carlotta Galeone, Gregorio Milani, Emilio Fossali, Paola Marchisio, Nicola Principi

**Affiliations:** 1 Department of Maternal and Pediatric Sciences, Università degli Studi di Milano, Fondazione IRCCS Ca' Granda Ospedale Maggiore Policlinico, Milan, Italy; 2 Department of Epidemiology, Istituto di Ricerche Farmacologiche "Mario Negri", Milan, Italy

## Abstract

This study evaluated the efficiency of pediatric mid-turbinate nasal flocked swabs used by parents in 203 children aged 6 months to 5 years with signs and symptoms of respiratory disease. Two nasal samples were collected from each child in a randomised sequence: one by a trained pediatrician and one by a parent. The real-time polymerase chain reaction influenza virus detection rates were similar in the samples collected using the two methods (Cohen's kappa = 0.86), as were the cycle threshold values. In comparison with the pediatrician-collected samples, the sensitivity and specificity of the parental collections were respectively 89.3% (95% confidence interval [CI]: 77.8-100%) and 97.7% (95% CI: 95.5-100%), and the positive and negative predictive values were respectively 86.2% (95% CI: 73.7-95.1%) and 98.2% (95% CI: 96.4-100%). The children were significantly more satisfied with the parental collections (median values ± standard deviation, 1.59 ± 0.55 *vs *3.51 ± 0.36; p < 0.0001). These findings show that mid-turbinate nasal flocked swabs specifically designed for infants and children can be used by parents without reducing the influenza virus detection rate. Moreover, the direct involvement of parents significantly increases patient acceptance, thus simplifying collection and suggesting that this novel swab design should be considered for epidemiological surveys and vaccine efficacy studies.

## Finding

In order to monitor the circulation of infectious agents and evaluate the efficacy of specific vaccines, it is essential to be able to identify the viruses that cause respiratory diseases in infants and children [1-6], and the adequate collection of respiratory specimens is the first crucial step in obtaining reliable information [7-9]. Such specimens are usually collected in hospital by certified nurses, pediatricians or other medical doctors, but parents may find it troublesome having to go to a hospital every time a specimen needs to be taken from a child with respiratory infection as such diseases occur several times a year. Collecting respiratory secretions at home could overcome this, but traditional collection techniques (mainly nasopharyngeal aspiration and nasopharyngeal washing) are too complex, invasive and time-consuming to be used by untrained people [10-12].

It has been found that recently developed mid-turbinate nasal flocked swabs are as effective as these traditional methods [13-15], and simple enough to be used by adult patients themselves and the parents of children [13,16]. However, as experience with the parental collection of samples is very limited, we evaluated the efficiency of pediatric mid-turbinate nasal flocked swabs when used by parents.

The study involved all of the children aged between six months and five years who attended the Emergency Department of the University of Milan's Department of Maternal and Pediatric Sciences because of signs and symptoms of respiratory disease between 1 January 2008 and 28 February 2008. Only the children with known craniofacial abnormalities were excluded. The protocol was approved by the Ethics Committee of the Fondazione IRCCS Ca' Granda Ospedale Maggiore Policlinico, and written informed consent was obtained from the parents of the enrolled children.

**Figure 1:**
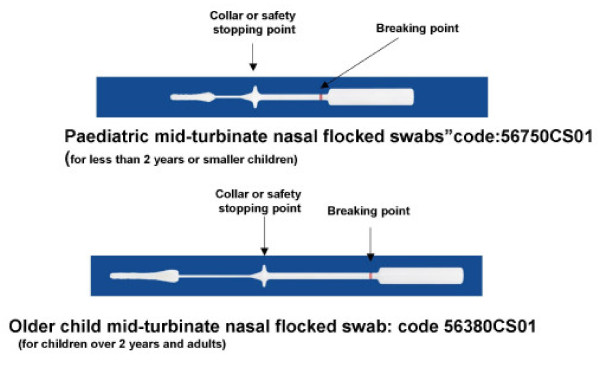
**Mid-turbinate nasal flocked swabs used by trained pediatrician or parents**.

Two nasal samples were collected in a randomised sequence from each child: one by a trained pediatrician (CT) and one by a parent. The pediatric mid-turbinated nasal flocked swabs (Copan, Brescia, Italy, code 56750CS01, suitable for children aged up to two years) and those for older children (code 56380CS01) have a collar respectively 2.5 and 5.5 centimeters along the swab shaft (Figure 1) that is large enough to prevent further insertion when it reaches the nostril. The pediatrician and a parent (who was first asked to read a very simply written and illustrated description of the procedure) each inserted a swab gently up to its collar and rotated it three times before placing it in viral transport medium to be delivered to the laboratory within three hours. The parents were then asked to describe their child's satisfaction with the two procedures using a a five-point scale (from 5 for "very satisfied" to 1 for "very unsatisfied"); an independent observer (PM) confirmed that the child's satisfaction was as reported by the parents. There was no refusal to participate, all of the children had two swabs taken (one by the pediatrician and one by a parent), and a satisfaction scale was completed for each.

As soon as they were delivered to the laboratory, each patient's paired samples were processed in parallel. Viral RNA was extracted from both swabs by means of a Nuclisens EasyMAG automated extraction system (Biomeriéux, Craponne, France), using phocine distemper virus (PDV) as an extraction control as previously described [6,9]. All of the real-time polymerase chain reactions (PCRs) were set up as singleplex PCRs in a total volume of 25 μL, using the Taqman Universal Master mix (Applied Biosystems, Foster City, CA, USA), 200-800 nM of primers, 100 nM of TaqMan probe and 10 μL of cDNA template, and the products were amplified using the ABI 7900HT Fast Real-Time PCR System (Applied Biosystems) and standard cycling parameters. The primer-probe sets were: influenza A, sense AAGACCAATCCTGTCACCTCTGA, antisense CAAAGCGTCTACGCTGCAGTCC, probe fam-TTTGTGTTCACGCTCACCGTGCC-bhq1; influenza B, sense GAGACACAATTGCCTACCTGCTT, antisense TTCTTTCCCACCGAACCAAC, probe tet-AGAAGATGGAGAAGGCAAAGCAGAACTAGC-eclipse; PDV, sense CGGGTGCCTTTTACAAGAAC, antisense TTCTTTCCTCAACCTCGTCC, probe vic-ATGCAAGGGCCAATTCTTCCAAGTT-bhq1. Influenza A and B RNA were quantified relatively; the criterion for a positive reaction was a cycle threshold (CT) of <40 cycles.

The findings relating to the specimens collected by the parents and pediatricians were compared using SAS version 9.1 software (SAS Institute, Cary, North Carolina). The categorical data were compared between groups using the χ^2 ^test or Fisher's test; the other between-group comparisons were made using Wilcoxon's signed-rank test, a non-parametric test for paired samples. P values of 0.05 or less were considered statistically significant.

The mean age ± standard deviation (SD) of the 203 recruited children was 1.99 ± 2.96 years: 103 (50.7%) were younger than two years, and the specimens were taken using the smaller swabs. Table 1 shows the detected influenza viruses. Thirty-two children (15.8%) were positive for influenza: the paired samples were both positive in 25 cases (12.3%), only the samples collected by the pediatrician were positive in three cases (1.5%), and only the samples collected by a parent were positive in four cases (2.0%). The influenza virus detection rates were similar in the samples collected using the two methods (Cohen's kappa = 0.86): in comparison with the pediatricians, the sensitivity and specificity of the parental collections were respectively 89.3% (95% confidence interval [CI]: 77.8-100%) and 97.7% (95% CI: 95.5-100%), and the positive and negative predictive values were respectively 86.2% (95% CI: 73.7-95.1%) and 98.2% (95% CI: 96.4-100%).

**Table 1:** Influenza viruses detected in mid-turbinate nasal flocked swabs collected from 203 children with influenza-like illness.

	No. of samples in which virus was detected			
	
Virus	Positive after pediatrician and parental collection	Positive after pediatrician collection alone	Positive after parental collection alone	Total number of positive samples
Influenza A	5 (2.5%)	0 (0.0%)	0 (0.0%)	5 (2.5%)
Influenza B	20 (9.8%)	3 (1.5%)	4 (2.0%)	27 (13.3%)
Total	25 (12.3%)	3 (1.5%)	4 (2.0%)	32 (15.8%)

No significant difference between the two methods of collection.

Table 2 summarises the CT values in the paired positive samples, which show that similar amounts of viruses were detected in the samples collected using the two methods. However, the children were significantly more satisfied with the parental collections (mean values ± SD, 1.59 ± 0.55 *vs *3.51 ± 0.36; p < 0.0001). The detection and satisfaction rates were similar regardless of the patients' age.

**Table 2:** Cycle threshold (CT) value in the paired samples positive after both pediatrician and parental collection.

Virus	CT value	
	
	Pediatrician collection	Parental collection
Influenza A (n = 5)	29.77 ± 5.45	29.17 ± 4.68
Influenza B (n = 20)	28.59 ± 3.82	29.43 ± 4.27

Mean values ± standard deviation (SD). No significant difference between the two methods of collection.

Our findings demonstrate that mid-turbinate nasal flocked swabs specifically designed for infants and children can be used by parents without reducing influenza virus detection rates. The number of influenza-positive nasal swabs and the CT values were similar in the samples collected by the pediatrician and parents. Furthermore, the direct involvement of parents significantly increased the patient's acceptance of the procedure and thus simplified collection.

These results suggest that, when an early evaluation of the viral etiology of a respiratory tract infection is needed, parents can collect respiratory secretions at home using pediatric mid-turbinate nasal flocked swabs. This has a number of advantages. First of all, if the child is included in an epidemiological survey or vaccine efficacy study, parental collection reduces the risk of losing the sample when respiratory episodes occur. Secondly, the samples can be obtained immediately after the onset of the first signs and symptoms, thus favouring the identification of the infectious agent and aiding treatment decision making after a pediatrician's visit. Thirdly, it reduces family organisational problems and the children's emotional involvement.

However, in order to make the most of such advantages, appropriate swabs specifically designed for infants and young children need to be used because the shafts of adult swabs are too long, and their tips are too big. Specifically prepared mid-turbinate nasal flocked swabs with a collar that prevents them from being inserted so deeply that they come into possibly painful contact with inflamed structures are safe and well tolerated, and can therefore be recommended in routine practice.

We do not know why the paired samples of seven children were not both positive, but only those taken by the pediatrician in three cases, and only those taken by a parent in four. There were no differences in CT values suggesting less virus and lower sensitivity, and no differences in the timing of the collections or in the age or weight of the children. In any case, the detection rates of the two collection methods were similar, and the sensitivity, specificity and positive and negative predictive values were high.

One limitation of this study is that, although the parents collected the respiratory secretions without any particular assistance, they were in our hospital and probably felt more confident knowing that professional help was on hand if needed; it is possible that they may have found it more difficult at home or that the sampling would have been less precise. To reduce these risks, it seems reasonable to suggest that they should be instructed by their child's pediatrician and that an illustrated explanation with details concerning specimen storage and transportation should be included in the package insert. Moreover, the study population was small and only influenza viruses were evaluated.

However, although further studies of larger populations designed to detect other respiratory viruses would strengthen our conclusions, we suggest that this novel swab design would be useful for epidemiological surveys or vaccine efficacy studies.

## List of abbreviations

CT: cycle threshold; CI: confidence interval; PDV: phocine distemper virus; PCR: polymerase chain reaction; SD: standard deviation.

## Competing interests

The authors declare that they have no competing interests.

## Authors' contributions

SE and NP designed the study and co-wrote the manuscript.  CGM, CD and AV carried out the real-time PCR.  CT collected the swabs.  CG performed the statistical analysis.  GM, EF and PM examined the patients.  All authors read and approved the final manuscript.
